# Long-term experience in treatment of acute promyelocytic leukemia in Mexican children in a tertiary care hospital

**DOI:** 10.3389/fonc.2023.1286106

**Published:** 2023-11-07

**Authors:** Marco Antonio Murillo-Maldonado, Paulina González Galván, Israel Parra Ortega, Félix Gaytán Morales, Luis Juárez Villegas, Elisa Dorantes Acosta

**Affiliations:** ^1^ Myeloid Leukemia Clinic, Federico Gómez, Children’s Hospital of Mexico, Mexico City, Mexico; ^2^ Laboratory of Molecular Biology, Federico Gómez, Children’s Hospital of Mexico Federico Gómez, Mexico City, Mexico; ^3^ Hematopoietic Stem Cell Transplant Unit, Federico Gómez, Children’s Hospital of Mexico Federico Gómez, Mexico City, Mexico; ^4^ Department of Hemato-Oncology, Federico Gómez, Children’s Hospital of Mexico Federico Gómez, Mexico City, Mexico; ^5^ Leukemia Cell Research Biobank, Federico, Gómez Children’s Hospital of Mexico, Mexico City, Mexico

**Keywords:** acute promyelocytic leukemia, ATRA, acute myeloid leukemia, cancer survival, children

## Abstract

**Introduction:**

Acute promyelocytic leukemia (APL) is a rare myeloid leukemia subtype affecting adult and pediatric populations. APL constitutes 15-20% of all childhood AML in Latin America, compared to 7% in the non-Latino population. This leukemia has unique characteristics, such as its association with chromosomal translocations involving the retinoid acid receptor α (RARA) gene on chromosome 17. In addition, APL is also distinct from other AML subtypes due to its response to all-trans-retinoic acid (ATRA), which induces terminal granulocytic differentiation of blasts. Overall 5-year survival rates are generally reported to be greater than 80%.

**Materials and methods:**

A study was conducted from January 2008 to December 2022 applying the IC-APL 2006 treatment protocol. This case series reports the clinical results of 22 children with APL. In all cases, the diagnosis was made by bone marrow aspiration and evaluation of the t(15:17) or t(11:17) transcripts.

**Results:**

We identified 22 patients with APL, of whom 10 were female and 12 were male. Twelve patients debuted with coagulation abnormalities. The doses of anthracyclines varied according to the risk, with an average of 496.8 mgm^2^. The cardiological assessment was performed before and after chemotherapy, finding 2/22 patients with moderate sisto-diastolic dysfunction and one with mild pulmonary insufficiency at the end of treatment. There were 6/22 patients with complications related to ATRA treatment, the most frequent being *pseudotumor cerebri*. All complications were transitory and treated immediately without complications. In this series of cases, an overall survival of 90.6% and a relapse-free survival of 90.6% were recorded. The follow-up mean was 9.1 ± 3.8 years.

**Conclusion:**

APL is a highly curable disease when combined with ATRA and anthracyclines. In this series of cases, good long-term results were observed with the IC-APL 2006 protocol. However, in Latin America, the availability of drugs such as arsenic trioxide as the first line of treatment is an unresolved challenge.

## Introduction

APL is a particular type of acute myeloid malignancy that is commonly characterized by the translocation t (15; 17)(q24.1;q21.2) and the resultant PML-RARA fusion gene (1). Since the protocolized use of all-trans retinoic acid (ATRA) in the 1980s and arsenic trioxide (ATO) in the 1990s, the outcomes of APL patients have improved substantially (2).

Over the last 60 years, APL has been recognized as the most malignant to the most curable form of acute leukemia (3, 4).

Even the epidemiology of lymphoid neoplasms has been widely studied, revealing that acute lymphoid leukemias are more frequent in the Latin American population (5, 6). On the other hand, myeloid malignancies are less studied. Data available from cross-sectional studies of APL patients in Latin America are scarce. Population-based information is not available due to inaccurate registries.

Douer et al. were the first to report specific features of APL in patients with ‘Latin’ and ‘non-Latin’ ancestry. The data suggested that the Latino population had a higher proportion of APL among all AML diagnoses, which reached 37.5% compared to 6.4% in the non-Latino population (7, 8).

APL is considered one of the most malignant forms of AML if it is not diagnosed and treated on time, because of the high rates of coagulopathy at diagnosis that can cause patient death (9).

The PETHEMA and GIMEMA (PETH/GIM) cooperative group identified risk factors associated with relapse and developed a predictive model based on white blood cell count (WBC) and platelet count (PLT) at diagnosis. This model was capable of stratifying patients into low risk (WBC < 10 x 10^9^/L/PLT > 40 x 10^9^/L), intermediate risk (WBC < 10 x 10^9^/L/PLT < 40 x 10^9^/L), and high risk (WBC > 10 x 10^9^/L PLT < 40 x 10^9^/L) groups (10).

However, patients who underwent treatment experienced impressive changes. First, the introduction of ATRA (all-trans retinoic acid) was added to chemotherapy protocols and led to survival rates above 90%, first applied in adults and later on replicating survival rates in children (11).

These regimens were associated with important morbidities, such as myelosuppression, secondary neoplasms, and anthracycline-related cardiomyopathy (12, 13).

Another substantial change in treatment was the introduction of arsenic trioxide (ATO), leading to entirely chemotherapy-free protocols in adults (14).

Novel treatment regimens for patients with high-risk APL, which included limited use of anthracycline (during induction therapy only) without other cytotoxic chemotherapy and shortened treatment duration without the use of maintenance therapy, set a new clinical therapeutic standard for childhood APL (14).

## Materials and methods

From January 2008 to December 2022, 156 files were collected with pediatric patients diagnosed with AML, of which 25 referred to a diagnosis of APL, three files were discarded, two because the diagnosis of APL was not corroborated, and one due to loss of follow-up in our institution.

Of the 22 patients who entered this study, primary caregivers signed informed consent forms at the start of treatment. Patients were diagnosed according to morphological and molecular criteria (15). Bone marrow samples were collected, morphologically evaluated and processed for PML-RAR alpha rearrangement using reverse transcriptase. In cases where the PML-RAR alpha transcript could not be detected, tests for the PZLF-RARa transcript were performed. Immunophenotypic analyses were systematically performed at the time of diagnosis and in cases of relapse. The remission induction response was assessed according to the recently revised criteria by molecular remission, and relapse was defined as the disappearance and reappearance of positive RT-PCR tests for the PML-RAR alpha fusion transcript.

Relapse risk groups were defined as PETHEMA and GIMEMA (PETH/GIM) cooperative groups to identify risk factors associated with relapse and develop a predictive model based on white blood cell count (WBC) and platelet count (PLT) at diagnosis. This model was capable of stratifying patients into low risk (WBC < 10 x 10^9^/L/PLT > 40 x 10^9^/L), intermediate risk (WBC < 10 x 10^9^/L/PLT < 40 x 10^9^/L), and high risk (WBC > 10 x 10^9^/L and PLT < 40 x 10^9^/L) groups (10).

Hematologic toxicity was graded according to Common Terminology Criteria for Adverse Events (CTCAE) Version 5 (16).

The patients had no cardiac contraindications to anthracycline chemotherapy and had serum creatinine levels < 3 times the normal upper limit and serum alanine aminotransferase/aspartate aminotransferase (ALT/AST) levels < 3 times the upper normal limit.

Coagulopathy was considered a defect in any of the normal hemostatic components (vasculature, platelets, coagulation factors, and fibrinolytic proteins) (17).

Supportive PLT transfusions were administered in the presence of bleeding with or without laboratory signs of severe coagulopathy or if the PLT count was < 50 x 10^9^/L. Blood cell units were transfused to maintain hemoglobin at levels > 8 g/dL. Treatment for differentiation syndrome involved the use of intravenous dexamethasone at a dose of 10 mg b.i.d. for a minimum of 3 days (12). The febrile episodes were treated according to the Guidelines for the Management of Fever and Neutropenia in Children with Cancer and Hematopoietic Stem-Cell Transplantation Recipients (18).

**Figure 1:**
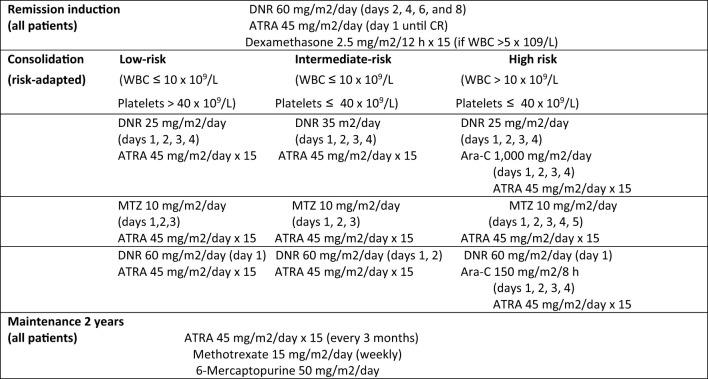
ASH International Committee Acute Promyelocytic Leukemia Protocol (IC-APL2006). ATRA: all-trans retinoic acid. DNR, daunorubicin; MTZ, mitoxantrone; Ara-C, cytarabine; CR, complete remission.

The treatment protocol comes from a consortium titled the International Consortium on Acute Promyelocytic Leukemia (IC APL) by The American Society of Hematology (19) (**Figure 1**).

APL specialists from numerous countries have contributed to the IC APL. This protocol is similar to the PETHEMA 2005 protocol but idarubicin is replaced with daunorubicin. The chemotherapy protocol consisted of administering ATRA and daunorubicin in the remission induction phase, followed by 3 cycles of chemotherapy in the consolidation phase and two years of maintenance (**Table 1**).

**Table 1:** General characteristics of the patients and clinical outcome.

Patient number	Age range (2-5 years old)	Age range (6-12 years old)	Age range (13-18 years old)	Gender	Morphology	WBC x 10^9^/L	Risk group *	Coagulopathy	Transcript PML RAR α	Current status
1			X	Female	Hypogranular	13,200	3	No	+	Surveillance
2		X		Male	Hypogranular	23,800	3	No	+	Surveillance
3	X			Male	Hypergranular	12,300	3	Yes	+	Surveillance
4		X		Male	Hypergranular	2,700	2	Yes	+	Relapse, after 60 months of diagnosis, under remission and under surveillance.
5		X		Female	Hypogranular	15,600	3	Yes	+	Surveillance
6		X		Male	Hypergranular	3,400	2	No	+	Surveillance
7	X			Male	Hypergranular	12,900	3	No	+	Surveillance
8		X		Male	Hypergranular	4,500	2	Yes	+	Surveillance
9		X		Male	Hypergranular	10,300	3	Yes	+	Surveillance
10		X		Female	Hypergranular	5,300	2	Yes	+	Surveillance
11		X		Male	Hypergranular	1,600	2	No	+	Surveillance
12	X			Female	Hypergranular	36,300	3	Yes	–	Surveillance
13		X		Male	Hypergranular	5,900	2	No	+	Surveillance
14	X			Female	Hypergranular	5,300	2	No	+	Surveillance
15		X		Female	Hypergranular	1700	1	No	+	Surveillance
16		X		Female	Hypergranular	3,000	1	No	+	Surveillance
17		X		Male	Hypergranular	4,000	1	Yes	+	Relapsed, after 14 months from diagnosis and died because infectious complications
18		X		Female	Hypergranular	600	2	Yes	+	Surveillance
19		X		Female	Hypergranular	1180	2	No	+	Surveillance
20		X		Female	Hypergranular	7,000	2	Yes	+	Surveillance
21			X	Male	Hypergranular	16,000	2	Yes	+	Surveillance
22		X		Male	Hypergranular	12,000	2	0Yes	+	Died because of bleeding complications during remission induction

*Relapse risk groups were defined as PETHEMA and GIMEMA (PETH/GIM) cooperative groups (10). Low risk =1, Intermediate risk =2, and high risk =3.

Relapses were confirmed by morphology and molecular investigations. Patients were tested for t(15;17) by RT‐PCR. At suspected relapse evaluation, a diagnostic lumbar puncture was performed with adequate blood product cover as required. After the diagnosis of relapse, patients received induction treatment with a combination of ATRA and anthracycline.

Cardiac function monitoring was performed with an echocardiogram at two points, at diagnosis, before first anthracycline, and at the end of maintenance.

The Kaplan-Meier curve was used to estimate the survival function from censored data.

We calculated survival rates of our specific population over time, and relapse-free survival rates.

## Results

Data were obtained from 22 patients over 14 years; the mean age at diagnosis was 8.6 years. Ten patients were female, and twelve were male.

The range of platelets at diagnosis was 4,000 to 116,000 x 10^9^ L (**Table 1**).

Twelve patients debuted with coagulation abnormalities. In one case, it was fatal since one patient died (Case 22) in the induction of remission due to bleeding in the brain parenchyma.

Three patients were classified as low risk, 12 as intermediate risk, and seven as high risk.

In all cases, bone marrow aspiration was performed at diagnosis and smear review for morphology, immunophenotype analysis, and detection of PML-RAR alpha rearrangement using reverse transcriptase.

Only one case (Case 12) found that although the morphological diagnosis was compatible with APL, we could never have a positive transcript for PML-RAR alpha or PZLF-RARa transcript. In this case, other transcripts not associated with APL were also analyzed, and all were negative.

In all patients, an echocardiogram was performed at the beginning to assess the use of anthracyclines; there was no contraindication for the start of treatment. The dose of anthracyclines was adjusted to the clinical risk and, on average, was 496.8 mgm^2^. One patient did not receive anthracyclines since the patient died a few days after diagnosis (Case 22).

In three cases, we found posttreatment cardiac changes, which were associated with the use of anthracyclines (Cases 2, 3, and 10).

The cardiac alterations found in two to twenty-two patients were moderate sisto-diastolic dysfunction (cases 2 and 3) and mild pulmonary insufficiency at the end of treatment (case 10) (**Table 2**).

**Table 2:** Cardiac function and complications secondary to the use of ATRA.

Patient number	Initial Cardiological Evaluation	Cardiologic Assessment End of treatment	Cumulative dose of anthracyclines (mgm^2^)	Complications associated with ATRA
1	Normal LVEF 70%	Normal LVEF 67%	450	Pseudotumor cerebri
2	Normal LVEF 86%	Moderate systodiastolic dysfunction LVEF 70%	450	Skin rash
3	Normal LVEF 78%	Disfuncion systodiastolic ventricular. LVEF 70%	450	None
4	Normal LVEF 82%	Normal LVEF 74%	900	Pseudotumor cerebri
5	Normal LVEF 65%	Normal LVEF 64%.	450	None
6	Normal LVEF 74%	Normal LVEF 69%	530	Differentiation syndrome
7	Normal LVEF 75%.	Normal LVEF 60%.	450	None
8	Normal LVEF 72%	Normal LVEF 61%.	530	Pseudotumor cerebri
9	Normal LVEF 70%	Normal LVEF 65%	450	None
10	Normal LVEF 64%	LVEF 56%. Normal biventricular function. Mild pulmonary insufficiency	530	None
11	Normal LVEF 65%	Not found	350	None
12	Normal LVEF 63%	Normal LVEF 60%	450	Differentiation syndrome
13	Normal LVEF 58%	Normal LVEF 56%	530	None
14	Normal LVEF 74%	Normal LVEF 61%	530	None
15	Normal LVEF 67%	Normal LVEF 64%	430	None
16	Normal LVEF 68%. Mild tricuspid insufficiency	Normal LVEF 63%. Mild tricuspid insufficiency	430	None
17	Reported as Normal LVEF	Not found	900	None
18	Normal LVEF 67%	Not found	530	None
19	Normal LVEF 62%	Not found	530	None
20	Normal LVEF 59%.	Not found	530	None
21	Normal LVEF 72%.	Not found	530	None
22	Reported as Normal LVEF	Not found	—	None

LVEF, left ventricular ejection fraction.

Cases 2 and 3 corresponded to high risk, and case 10 corresponded to intermediate risk. The three patients are under clinical surveillance with no signs of deterioration in their functional class.

There were 6/22 patients with complications related to ATRA treatment. All complications occurred during induction to remission, and the most frequent complication was *pseudotumor cerebri* in 4 cases. Second, 3 patients had differentiation syndrome, and one patient presented skin toxicity with a generalized rash attributed to ATRA (**Table 2**).

All complications were transient and treated immediately without complications.

ATRA was never eliminated from treatment.

Two patients relapsed (Cases 4 and 17), the first with intermediate risk and the second with low risk.

Both relapses were confirmed by morphology and molecular investigations. Both patients were positive for t(15;17) by RT‐PCR. At suspected relapse evaluation, a diagnostic lumbar puncture was performed on these two patients with adequate blood product cover as required. After the diagnosis of relapse, patients received induction treatment with a combination of ATRA and anthracycline (19).

In case 4, relapse occurred 60 months after diagnosis; remission was again induced, and he is currently under oncological surveillance with molecular remission.

In Case 17, the patient relapsed 14 months after diagnosis, remission could not be achieved, and he died of infectious complications.

In the case of the two deaths (Cases 17 and 22), the first patient died during relapse, and the second case corresponded to a patient who died in the induction of remission with hemorrhagic complications in the central nervous system in the intensive care unit. Only ATRA was administered to this patient, who died a few days after admission.

**Figure 2:**
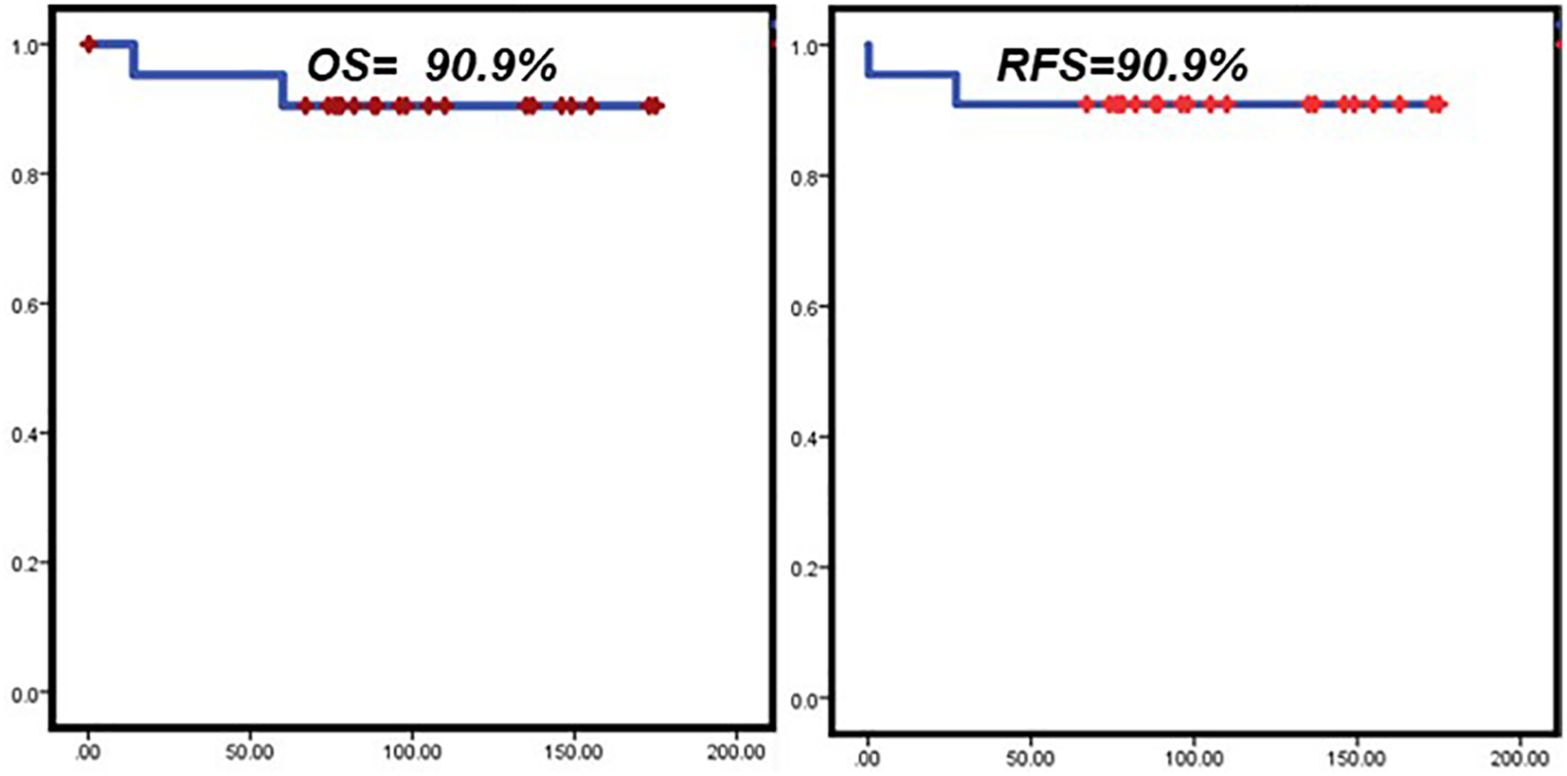
Overall Survival and Relapse free survival in study patients. Kaplan-Meier curves. Overall survival rates (left), and relapse-free survival rates over time (right) were calculated. OS, Overall Survival; RFS, Relapse free survival.

In this series of cases, an overall survival of 90.6% and a relapse-free survival of 90.6% were achieved (**Figure 2**).

The follow-up mean was 9.1 ± 3.8 years.

## Conclusion

Mexico is immersed in Latin America. The Latin America region presents an epidemiological challenge due to its population’s heterogeneity and socioeconomic inequality. Cancer treatment is expensive and prolonged; this area of ​​the world was hit the hardest by the pandemic (20). The region’s economy grew by 6.2% in 2021 and only expanded 2.1% in 2022. According to ECLAC, the emerging economies would only summarize these trends in 2025.” (21).

Recent studies (22) have found that childhood cancer in Mexico is affected by a mixture of factors such as molecular epidemiology, late/low precision diagnosis, limited access to treatment, toxicity associated with therapy, continuous exposure to environmental risk factors, and all of this cooperating to low rates of survival in low-to-medium-income countries.

Regarding ATO, it is not yet used as a first line in patients with APL in Mexico, and access to the most expensive anthracycline drugs is a challenge for public hospitals.

It is also important to mention that hospital infrastructure is critical in this type of leukemia, and access to blood banks and transfusion products is essential to avoid early mortality (23).

In our study, a series of cases of patients with APL is analyzed, constituting 14.1% of the total AML population, consistent with the international literature that indicates the higher frequency of this subtype of myeloid leukemia in Latin American countries (7, 8).

This long-term study shows a number of pediatric cases treated according to a protocol proposed by the International Consortium on Acute Promyelocytic Leukemia (IC APL) by The American Society of Hematology, where it is shown that patients benefit from the establishment of protocols adapted to the sociodemographic and economic situation; for example, anthracyclines such as idarubicin are substituted with daunorubicin, which is less expensive. Adhering to standardized protocols benefits overall and relapse-free survival. Our overall survival is reported to be above 90%, which is adequate.

In meta-analysis results comparing treatment regimens for patients with myeloid leukemia, results have been observed where regimens that include idarubicin have a better effect of inducing remission than those that include daunorubicin. The difference in adverse events and cytogenetic subgroup analysis between the idarubicin and daunorubicin groups were not statistically significant. Therefore, it is concluded that the idarubicin regimen can be applied in the clinic as an induction regimen for AML (24).

Subclinical cardiotoxicity or preclinical cardiotoxicity refers to the initial phase of this cardiomyopathy when the disease is not yet clinically manifested. Given that the current diagnosis of cardiotoxicity continues to be based on the appearance of heart failure symptoms or a decrease in the left ventricular ejection fraction (LVEF) and taking into account the existing interobserver variability in the determination of LVEF, the incidence of cardiotoxicity may vary depending on the type of antineoplastic treatment and the type of detection system used to establish the diagnosis. The use of highly sensitive cardiac troponin I and new echocardiographic parameters such as strain/strain rate and new biomarkers capable of identifying patients at risk for cardiac disease may help establish an early diagnosis of the disease (25).

In our study, echocardiographic estimation revealed that 13.6% of the patients had secondary damage to anthracyclines, which is high when compared to the data found in the literature.

In a retrospective analysis by Von Hoff et al. (26), the percentage of patients who had left ventricular dysfunction detected by echocardiographic estimation of LVEF with a cumulative dose of doxorubicin 400 mgm^2^ was 3% and increased to 7% with 550 mgm^2^ and 18% with 700 mgm^2^.

International clinical trials have found excellent patient survival rates, suggesting that ATRA/arsenic trioxide therapy is beneficial for treating pediatric patients with standard-risk and high-risk APL. The study results confirmed that pediatric patients with standard-risk APL could be safely treated with ATRA/arsenic trioxide therapy and achieve results similar to those in adult patients, for whom this treatment has become the preferred regimen. This will limit the use of anthracyclines and improve the morbidity rates associated with cardiotoxicity in the medium and long term (14). In Latin American countries, clinical trials support the use of new drugs that have been shown to be effective in other parts of the world and are also adopted as first-line treatments.

In a study with 509 patients carried out by Sanz et al. (12), it was observed that the most significant proportion of patients were classified as intermediate risk (57.9%), high risk (21.8%) and low risk (20.2%).

In our study, although the number of patients is smaller, the highest proportion of patients was intermediate risk (54.5%), followed by high risk (31.8%) and low risk (13.6%).

Regarding ATRA toxicity in the study patients, we report the resolution of all those that occurred, which shows that this therapy is very well tolerated in our population.

In this work, the most frequent complication was *pseudotumor cerebri* in 4 cases.

Pseudotumor cerebri (PTC) has frequently been described in the literature as an adverse effect of ATRA therapy, usually in cross sectional reports.

Coombs et al. reported the series of 240 patients in which there was a clinical suspicion for PTC.Probable PTC occurred in 1.7% of patients who received ATRA during induction and/or maintenance chemotherapy. In agreement with previous reports, the incidence of PTC in APL patients receiving ATRA was higher in the pediatric population.

One possibility is that the diagnostic criteria are different, so an important point to consider is standardizing criteria to compare the data reported in the literature (27).

The rarest complication was skin toxicity, previously described in other case reports with a broad spectrum of clinical manifestations (28).

We didn’t find a link between the two patients that relapsed, maybe because of the short number of relapses in this work.

While survival is the most critical outcome of cancer treatment, it will be essential that in this type of disease, where high cure rates are obtained, follow-up studies are generated that evaluate other improvements in patient survival, such as quality of life.

This work shows that despite the existing limitations in a country from Latin America, such as the difficulties in offering treatment with broader options from required medications, it is possible to obtain adequate results by making the correct adjustments based on scientific evidence.

## Data availability statement

The raw data supporting the conclusions of this article will be made available by the authors, without undue reservation.

## Ethics statement

The studies involving humans were approved by Ethics and Research Committee of the Children’s Hospital of Mexico Federico Gomez. The studies were conducted in accordance with the local legislation and institutional requirements. Written informed consent for participation in this study was provided by the participants’ legal guardians/next of kin. Written informed consent was obtained from the individual(s) for the publication of any potentially identifiable images or data included in this article.

## Author contributions

MM-M: Conceptualization, Investigation, Writing – original draft. PG: Writing – original draft. IP: Investigation, Writing – original draft. FG: Conceptualization, Writing – original draft. LJ: Investigation, Writing – original draft. ED: Conceptualization, Formal Analysis, Investigation, Methodology, Supervision, Writing – original draft, Writing – review & editing.
